# Mentoring during Medical School and Match Outcome among Emergency Medicine Residents

**DOI:** 10.5811/westjem.2015.9.27010

**Published:** 2015-11-12

**Authors:** Erin Dehon, Margaret H. Cruse, Brandon Dawson, Loretta Jackson-Williams

**Affiliations:** University of Mississippi Medical Center, Department of Emergency Medicine, Jackson, Mississippi

## Abstract

**Introduction:**

Few studies have documented the value of mentoring for medical students, and research has been limited to more subjective (e.g., job satisfaction, perceived career preparation) rather than objective outcomes. This study examined whether having a mentor is associated with match outcome (where a student matched based on their rank order list [ROL]).

**Methods:**

We sent a survey link to all emergency medicine (EM) program coordinators to distribute to their residents. EM residents were surveyed about whether they had a mentor during medical school. Match outcome was assessed by asking residents where they matched on their ROL (e.g., first choice, fifth choice). They were also asked about rank in medical school, type of degree (MD vs. DO), and performance on standardized tests. Residents who indicated having a mentor completed the Mentorship Effectiveness Scale (MES), which evaluates behavioral characteristics of the mentor and yields a total score. We assessed correlations among these variables using Pearson’s correlation coefficient. Post-hoc analysis using independent sample t-test was conducted to compare differences in the MES score between those who matched to their first or second choice vs. third or higher choice.

**Results:**

Participants were a convenience sample of 297 EM residents. Of those, 199 (67%) reported having a mentor during medical school. Contrary to our hypothesis, there was no significant correlation between having a mentor and match outcome (r=0.06, p=0.29). Match outcome was associated with class rank (r=0.13, p=0.03), satisfaction with match outcome (r= −0.37, p<0.001), and type of degree (r=0.12, p=0.04). Among those with mentors, a t-test revealed that the MES score was significantly higher among those who matched to their first or second choice (M=51.31, SD=10.13) compared to those who matched to their third or higher choice (M=43.59, SD=17.12), t(194)=3.65, p<0.001, d=0.55.

**Conclusion:**

Simply having a mentor during medical school does not impact match outcome, but having an effective mentor is associated with a more favorable match outcome among medical students applying to EM programs.

## INTRODUCTION

Mentoring has been associated with numerous benefits for individuals working in fields ranging from business to academic medicine. Among academic physicians, mentoring is associated with increased job satisfaction, higher salary, increased research productivity, and career advancement.^1^–^3^ Physicians with mentors were found to be 2.3 times more likely to be promoted than those without mentors.^4^ Mentoring has demonstrated similar benefits for medical trainees including both residents and medical students. Compared to non-mentored residents, mentored residents were twice as likely to state that they received excellent career preparation.^5^ A systematic review of mentoring programs for medical students revealed that having a mentor is associated with increased research productivity and interest in academic careers, enhanced well-being, and specialty choice for medical students.^6^

Despite the aforementioned benefits of mentoring for medical trainees, few studies, with the exception of those focused on research productivity, have examined quantifiable (vs. subjective) benefits of mentoring for medical trainees. Furthermore, few studies have examined the value of mentoring among medical students who enter emergency medicine (EM). The purpose of this study was to examine EM residents’ experience of mentorship during medical school and its relationship to match outcome. Our hypothesis is that EM residents who report having a mentor during medical school will be more likely to have matched to a residency program at the top of their rank order list (ROL).

## METHODS

Participants were recruited through the EM Association of Residency Coordinators listserv. An email with information about the study and a link to the survey was sent to the program coordinators who were asked to distribute the email to their EM residents. The institutional review board approved this study and a waiver of signed consent.

EM residents were surveyed about whether they had a mentor during medical school using the definition provided by Ramanan^5^: “an active partner in an ongoing relationship who helps you maximize your potential and achieve your personal and professional goals.” Residents also reported their rank in medical school, degree (MD vs. DO), location of medical school (U.S. or international) and performance on standardized tests. For the purpose of this study, we assessed match outcome by asking residents where they matched based on their ROL (e.g., first choice, fifth choice). Resident satisfaction with match outcome was measured using a five-point scale (very dissatisfied to very satisfied).

Residents who indicated having a mentor were directed to complete the Mentorship Effectiveness Scale (MES).^7^ The MES is a 12-item self-report measure designed to assess the overall effectiveness of mentoring. Each item describes behavioral characteristics of a mentor, which are rated using a five-point Likert-type scale (0=strongly disagree, 5=strongly agree) or “NA” if the item did not apply. Lastly, residents reported length of relationship with mentor, gender of mentor, and whether or not they still communicated with their mentor.

We used chi-square analyses to compare applicant characteristics (e.g., sex, United States Medical Licensing Examination [USMLE] score, rank in medical school) of those with and without mentors. Pearson correlations were conducted to examine the relationship between having a mentor and match outcome. We conducted post-hoc analysis using an independent sample t-test to compare differences in the MES score between those who matched to one of their top two choices vs. third or higher choice.

## RESULTS

The convenience sample was 297 EM residents; 59% (n=176) were male and 41% (n=121) were female. The majority were allopathic (79%, n=235) and U.S. graduates (93%, n=277). These characteristics are largely consistent with the National Resident Matching Program data.^8^ About two-thirds (67%) reported having a mentor during medical school. Males (66%, n=117) and females (67%, n=82) reported having a mentor during medical school. Of those with mentors, 76% (n=148) reported that their mentor was self-identified versus assigned by their school 24% (n=46). Most mentors were EM physicians (80%, n=159). Male mentors (72%, n=140) were more common than female mentors (28%, n=55). About half (55%, n=110) reported that they still communicated with their mentor.

A comparison of those with and without mentors is presented in Table 1. There was a significant association between type of degree and mentorship, χ^2^(1, n=297)=6.73, p<0.01 with the odds of having a mentor 2.1 times higher among those with an allopathic degree. There was also a significant association between location of medical school and mentorship, χ^2^ (1, n=297)=6.73, p<0.05. The odds of having a mentor were 3.4 times higher for those who attended a U.S. school.

Regarding match outcome, the majority of respondents reported matching to their first choice (n=176, 59%), followed by second (n=56, 19%), third (n=27, 9%), fourth (5%, n=15), fifth (2%, n=7), sixth (1%, n=4), seventh (1%, n=3), and 8^th^ or higher choice (3%, n=8). Contrary to our hypothesis, there was no significant correlation between having a mentor and match outcome (r=0.06, p=0.29). A nearly equal number of respondents with and without mentors matched to one of their top two choices (Table 1). Match outcome was significantly associated with class rank (r=0.13, p=0.03), satisfaction with match outcome (r= −0.37, p<0.001), and having an MD (vs. DO) (r=0.12, p=0.04). USMLE was not significantly associated with match outcome.

Among those with mentors, we used a t-test to compare MES scores among EM residents who matched to one of their top two choices to those who matched lower on their list. The MES score was significantly higher among those who matched to their first or second choice (M=51.31, SD=10.13) compared to those who matched lower on their ROL (M=43.59, SD=17.12), t(194)=3.65, p<0.001, d=0.55. Table 2 shows the descriptive statistics for each of the items on the MES. Residents who still communicated with their mentor were more likely to have matched to their first or second choice, χ^2^ (1, n=198)=10.79, p<0.01.

## DISCUSSION

Findings do not provide support for the hypothesized relationships between having a mentor during medical school and matching high on one’s rank list. Match outcome was more likely to be associated with other factors including class rank and type of degree (i.e., MD). Nonetheless, we did find a relationship between degree of mentorship effectiveness and match outcome. Specifically, we found that among EM residents with mentors, those who reported greater mentor effectiveness were more likely to match to their first or second choice. Taken together, these findings suggest having a supportive, motivating, and helpful mentor may be one of many factors that can increase an applicant’s chance of matching to one of their top choices.

This study also sheds light on the prevalence of mentoring among medical school students who enter EM. Two-thirds of the respondents reported having a mentor during medical school. This number is much higher than previous reports, which found the prevalence of mentorship among medical students to be 50%.^2^ It is unclear whether this finding reflects a genuine increase in mentoring, a higher prevalence of mentoring among students interested in EM, or some other anomaly. Given that the definitions of mentoring often vary from study to study, it is often difficult to make meaningful comparisons across studies. We also found that graduates of osteopathic and international graduates were less likely to have mentors compared to allopathic and U.S. graduates. It may be that mentoring programs are more prevalent in U.S. allopathic schools, although most respondents (76%) reported that their mentor was self-identified, not assigned by the medical school.

## LIMITATIONS AND FUTURE DIRECTIONS

First, a major concern is the relatively small sample size and use of a convenience sample. Furthermore, given the design of our study we were unable to include unmatched applicants which could have had a significant impact on the results. Second, these results are based on unverified self-report data. Future studies would benefit from the inclusion of other methods of data collection, such as a review of medical school records for verification of data. Third, the finding that most mentors were self-identified raises the question of whether students who seek out mentors have personal characteristics (e.g., motivation) that contribute to their success. Fourth, given the correlational nature of the study, it is impossible to determine the exact nature of the relationship between mentorship and match outcome. Future studies that incorporate pre and post designs and/or random assignment of students to mentors are needed to more fully examine the relationship between mentorship and match outcome. Lastly, although we found a significant difference between the MES scores for higher vs. lower matching students, more research is needed to verify the meaningfulness of these results.

## CONCLUSION

These results suggest that simply having a mentor during medical school does not impact match outcome but having an effective mentor is associated with a more favorable match outcome among medical students applying to EM programs.

## Figures and Tables

**Table 1:** Characteristics of residents with and without mentors during medical school.

Characteristic	Mentor (n=199)	No mentor (n=98)	Chi-square	P
Sex [n (%)]			0.05	0.82
Male	117 (58.8%)	59 (60.2%)		
Female	82 (41.2%)	39 (39.8%)		
USMLE score [n (%)]			3.27	0.35
181–210	18 (9.3%)	13 (13.7%)		
211–250	131 (67.5%)	59 (62.1%)		
>250	36 (18.6%)	15 (15.8%)		
Did not take USMLE	9 (4.6%)	8 (8.4%)		
Rank in medical school [n (%)]			2.50	0.65
Top sixth	42 (21.1%)	26 (27.1%)		
Top third	48 (24.1%)	25 (26.0%)		
Middle third	59 (29.6%)	27 (28.1%)		
Bottom third	11 (5.5%)	3 (3.1%)		
Not used by medical school	39 (19.6%)	15 (15.6%)		
Degree [n (%)]			6.73	0.009**
MD (allopathic)	166 (83.4%)	69 (70.4%)		
DO (osteopathic)	33 (16.6%)	29 (29.6%)		
Medical school location [n (%)]			6.72	0.01*
United States	190 (96.4%)	87 (88.8%)		
International	7 (3.6%)	11 (11.2%)		
Match outcome [n (%)]			0.71	0.39
1^st^ or 2^nd^ choice	158 (79.8%)	74 (75.5%)		
3^rd^ choice or higher	40 (20.2%)	24 (24.5%)		
Satisfaction with match [n (%)]			8.56	0.07
Very dissatisfied	6 (3.1%)	4 (4.2%)		
Dissatisfied	4 (2.1%)	1 (1.0%)		
Neutral	9 (4.6%)	4 (4.2%)		
Satisfied	33 (16.9%)	30 (31.3%)		
Very satisfied	143 (73.3%)	57 (59.4%)		

*USMLE,* United States medical licensing exam

* Significant at p<0.05.

** Significant at p<0.01.

**Table 2:** Descriptive statistics of mentor effectiveness and match outcome. Means for the Mentoring Effectiveness Scale items are based on a five-point scale from 0 (strongly disagree) to 5 (strongly agree). NA was an option and was coded as 0.

Variable	Mean	SD
My mentor was accessible.	4.38	0.95
My mentor demonstrated professional integrity.	4.66	0.64
My mentor demonstrated content expertise in my area of need.	4.47	0.90
My mentor was approachable and easy to talk with about concerns.	4.58	0.84
My mentor was supportive and encouraging.	4.58	0.86
My mentor provided constructive and useful critique of my work.	4.24	1.01
My mentor motivated me to improve my work product.	4.25	1.03
My mentor was helpful in providing direction and guidance on professional issues (e.g., networking).	4.00	1.26
My mentor answered my questions satisfactorily (e.g., timely response, clear, comprehensive).	4.47	0.93
My mentor was helpful in providing advice on work/school and personal life.	4.04	1.30
My mentor suggested appropriate resources (e.g., experts, contacts, source materials).	4.22	1.06
My mentor challenged me to extend my abilities (e.g., risk taking, try a new activity, draft a section of an article).	3.98	1.26
Total score	49.79	12.17

*SD,* standard deviation
